# Infrared microspectroscopic imaging of plant tissues: spectral visualization of *Triticum aestivum* kernel and Arabidopsis leaf microstructure

**DOI:** 10.1111/tpj.13031

**Published:** 2015-10-23

**Authors:** Frederick J Warren, Benjamin B Perston, Silvia P Galindez-Najera, Cathrina H Edwards, Prudence O Powell, Giusy Mandalari, Grant M Campbell, Peter J Butterworth, Peter R Ellis

**Affiliations:** 1 King's College London, Faculty of Life Sciences and Medicine, Diabetes and Nutritional Sciences Division, Biopolymers Group, LondonFranklin-Wilkins Building, 150, Stamford Street, London, SE1 9NH, United Kingdom; 2 ARC Centre of Excellence in Plant Cell Walls, Centre for Nutrition and Food Sciences, Queensland Alliance for Agriculture and Food Innovation, The University of QueenslandSt. Lucia, Brisbane, Queensland, 4072, Australia; 3 PerkinElmerChalfont Road, Seer Green, Buckinghamshire, HP9 2FX, United Kingdom; 4 Satake Centre for Grain Process Engineering, School of Chemical Engineering and Analytical Science, The University of ManchesterM13 9PL, Manchester, United Kingdom; 5 The Model Gut, Institute of Food Research, Norwich Research ParkColney Lane, NR4 7UA, Norwich, United Kingdom; 6 Department of Drug Science and Products for Health, University of MessinaVill. SS. Annunziata, 98168, Messina, Italy

**Keywords:** infrared microspectroscopy, hyperspectral imaging, wheat grain structure, *in vitro* digestion, *Triticum aestivum* L., *Arabidopsis thaliana*

## Abstract

Infrared microspectroscopy is a tool with potential for studies of the microstructure, chemical composition and functionality of plants at a subcellular level. Here we present the use of high-resolution bench top-based infrared microspectroscopy to investigate the microstructure of *Triticum aestivum* L. (wheat) kernels and Arabidopsis leaves. Images of isolated wheat kernel tissues and whole wheat kernels following hydrothermal processing and simulated gastric and duodenal digestion were generated, as well as images of Arabidopsis leaves at different points during a diurnal cycle. Individual cells and cell walls were resolved, and large structures within cells, such as starch granules and protein bodies, were clearly identified. Contrast was provided by converting the hyperspectral image cubes into false-colour images using either principal component analysis (PCA) overlays or by correlation analysis. The unsupervised PCA approach provided a clear view of the sample microstructure, whereas the correlation analysis was used to confirm the identity of different anatomical structures using the spectra from isolated components. It was then demonstrated that gelatinized and native starch within cells could be distinguished, and that the loss of starch during wheat digestion could be observed, as well as the accumulation of starch in leaves during a diurnal period.

## Introduction

*Triticum* spp. (wheat) is one of the most widely used food ingredients in the world. It is a key component of a number of staple foods, most obviously bread. Studies of the microstructure of wheat are crucial for understanding the functionality of wheat as a food ingredient, its potential use as a biofuel crop and how macronutrients in wheat-based foods are digested in the mammalian gastrointestinal tract (Shewry, 2009). For example, changes in the rate and extent of starch digestion in the human gut are known to influence postprandial glycaemia, large fluctuations of which are linked with an increased risk of developing cardiometabolic diseases (e.g. type–2 diabetes; Jenkins *et al*., 2002). Moreover, the extent of digestion of starch and other macronutrients has important implications for energy metabolism, and obesity risk and management.

The four major tissue fractions of a wheat kernel are: the endosperm (the starch-rich material used for making white flour); the pericarp-testa (the tough outer fruit and seed coats of the kernel, which form most of the bran); the germ (the lipid- and protein-rich embryo and commercial source of oil); and the aleurone, the single layer of cells between the endosperm and pericarp-testa that contains the majority of the micronutrient content of wheat, e.g. iron and B vitamins (Barron and Rouau, 2008; Barron *et al*., 2007; Harris *et al*., 2005).

Visualizing and quantifying these different components of the wheat grain during processing or digestion has been an important but challenging research question that has attracted attention since the 1970s. Early imaging work concentrated on measuring bran through its autofluorescence of ferulic acid (Fulcher *et al*., 1972), which is mainly located in the pericarp-testa layers. As well as allowing the branny layers to be visualized, ferulic acid can be quantified by reversed phase (RP)-HPLC to estimate the proportion of bran in milled wheat (Pussayanawin and Wetzel, 1987; Pussayanawin *et al*., 1988). More advanced chromatography approaches use a combination of RP-HPLC and GC to quantify the amino acid, sugar and phenolic acid contents of the separate wheat tissues (Jensen and Martens, 1983; Barron *et al*., 2007). Combining all of these biochemical markers together with others, such as micronutrient components, and using multivariate data analysis tools results in a method that can quantify endosperm, aleurone and pericarp-testa with high accuracy (Hemery *et al*., 2009). It is, however, a slow and laborious process, requiring a total of six separate analyses and necessitating an operator with expertise in ELISA, RP-HPLC, GC, chemical extraction and enzyme assay methods. Also, it is impossible to quantify the germ directly by these compositional methods. Hemery *et al*. (2009) estimated the nominal germ content by subtraction, but the proportion of germ in wheat grain (2–5% w/w) is lower than the cumulative errors in the assays used to identify the other three fractions, potentially leading to large errors in the estimation of the germ fraction. Critically, this method also reveals nothing about the spatial distribution of components within the wheat kernel, because the samples are homogenized prior to analysis.

Recently, mid-infrared spectroscopy has received increasing interest as a tool to study the chemical composition of wheat grains at the tissue level, using attenuated total reflectance Fourier transform infrared spectroscopy (ATR-FTIR). Mid-infrared spectroscopy has the advantage of measuring all of the organic chemical constituents of a sample simultaneously, allowing the constituent tissues of the wheat grain to be rapidly (within ∼30 s) and simultaneously quantified (Barron, 2011).

Infrared microspectroscopy has also been applied to the model plant *Arabidopsis thaliana* as a tool to allow the simultaneous analysis of multiple chemical components within the Arabidopsis leaf (Mazurek *et al*., 2013). This tool has the potential to allow the distribution of chemical compounds in Arabidopsis leaf tissues to be quantified with potentially subcellular resolution across tissues. Fixing and embedding leaf tissue prior to sectioning enables a further level of temporal resolution to be achieved, allowing diurnal variations in the chemical composition of plant tissues to be visualized at a subcellular level. Many metabolites undergo diurnal variations in the Arabadopsis leaf, the largest of which is the deposition of starch using carbon assimilated from photosynthesis during the light period, which is subsequently broken down and metabolized during the dark period (Zeeman *et al*., 2007). Carbon assimilated by photosynthesis in Arabidopsis is predominantly stored in the form of starch in chloroplasts. Alterations in the deposition and breakdown of this transient leaf starch in Arabidopsis can have dramatic effects on the growth and development of the plants, making it a key model system for understanding the mechanisms of the synthesis and degradation of starch in plants (Zeeman *et al*., 2007; Pfister *et al*., 2014).

Mid-infrared microspectroscopic imaging is a technique that allows spectral data to be collected from a sample with spatial resolution, generating a three-dimensional data cube, with *x*- and *y*–axes representing units of μm, and with the *z*–axis consisting of spectra collected at each coordinate. The results of analysis of the collected spectra can therefore be mapped in two dimensions to produce an image of the spatial distribution of chemical components across a sample. A number of different analysis methods can be applied to the spectra obtained, such as principal component analysis (PCA), correlation analysis or mapping absorption values at specific wavelengths. These approaches can allow the spatial locations of different tissues, with differing chemical composition, to be assessed without the need to use specific stains or combinations of stains. As the aleurone layer is only one cell thick, any imaging method requires better than cellular (20–50 μm) resolution to successfully identify all of the tissue types in the wheat kernel. Mid-infrared radiation has a wavelength of around 2.5–14.0 μm, with the most valuable chemical information for characterizing wheat components found at the longer wavelength end of the spectrum. Taking a Bragg resolution limit of approximately λ/2, a resolution of ∼5–7 μm should be possible. Studies in the 1990s and early 2000s have used synchrotron light sources for transmission-mode imaging (Wetzel *et al*., 1998). More recently, specially prepared sections on barium fluoride (BaF_2_) discs have been used for transmission-mode imaging with a conventional light source to image isolated wheat grain components and whole grain sections, to a resolution of 5–10 μm (Marcott *et al*., 1999; Barron *et al*., 2005). At this resolution it is possible to identify whole tissues, but individual cells are difficult to resolve, even after the cell contents are removed by sonicating tissue sections (Barron *et al*., 2005).

Recent advances in infrared microspectroscopic imaging have used a combination of a germanium (Ge) ATR crystal and a focal-plane array or linear-array detector to increase the spatial resolution that can be achieved. The high refractive index of the Ge crystal (4.0) reduces the resolution-limiting effect of diffraction, with the system used in the present study having a quoted spatial resolution of 3.1 μm for light with a wavelength of 5.8 μm in air (PerkinElmer 2006). A further useful property of the ATR sampling mode is that it does not require a transparent sample: absorption occurs by means of the sample interacting with the evanescent wave formed at the surface of the crystal, which has an effective penetration into the sample of less than 1 μm. The spatial resolution and signal-to-noise performance obtained in the present study allow the imaging of the chemical and physical structure of the different tissue fractions of the wheat kernel at cellular resolution. Such findings are expected to facilitate our understanding, at a microstructural level, of how the different parts of wheat are physically and chemically altered during mechanical (de-branning and milling) and thermal processing, and also during simulated digestion in a dynamic model of the human gastrointestinal tract.

In this paper we describe the use of ATR imaging using a Ge crystal with a Spotlight 400 system incorporating a linear-array detector to image hand-dissected isolated wheat grain tissues and sections through whole wheat grains. Imaging experiments were performed on hand-dissected and milled fractions of winter wheat varieties, Consort and Malacca, whereas durum wheat was imaged after hydrothermal processing, and before and after digestion, using an *in vitro* model of the human upper gastrointestinal tract. We also image fresh picked, as well as embedded and sectioned, Arabidopsis leaves at the end of dark and light periods, comparing the deposition of starch in the leaves, as well as the improvements in resolution that may be achieved through embedding samples prior to infrared microspectroscopic analysis.

## Results

### Hand-dissected isolated wheat components

**Figure 1:**
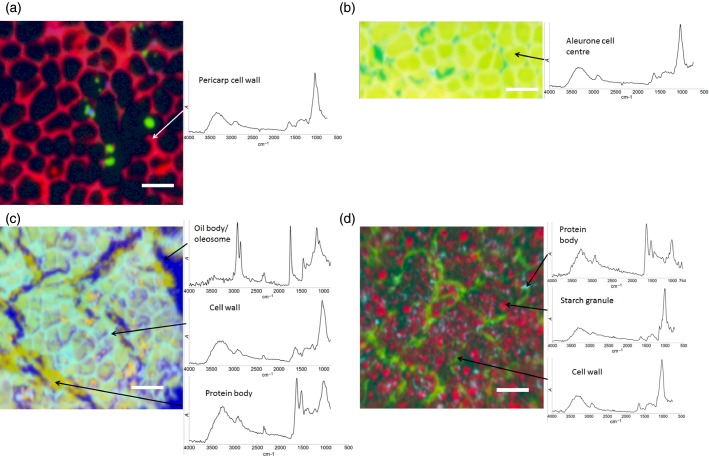
Images of hand-dissected wheat kernel tissues (Consort), shown as false-colour principal component analysis (PCA) images, with example spectra provided for regions of interest: (a) pericarp-testa; (b) aleurone; (c) germ; and (d) endosperm. Scale bars: 50 μm.

Images were obtained of hand-isolated pericarp-testa, aleurone, germ and endosperm (Figure1a–d, respectively) following isolation from the wheat grain, and without any embedding (the hand-dissected samples were small and offered smooth surfaces for imaging). Hyperspectral images were successfully obtained for each of the tissues, showing their differing structures and chemical compositions.

The pericarp-testa (Figure1a) can be seen to consist of a series of largely empty cells, with only the network of thickened cell walls (shown in red) being imaged by the microscope. The strong absorbance bands in the region 1000–1200 cm^−1^ of the spectrum shown in Figure1(a) are typical of the C–O stretching modes of the arabinoxylans, other hemicelluloses and cellulose, of which the pericarp-testa cell walls are composed. The green spheres may be stray starch granules that have adhered to the pericarp-testa cell walls, or may represent the small number of starch granules that occur naturally in the pericarp-testa.

**Figure 2:**
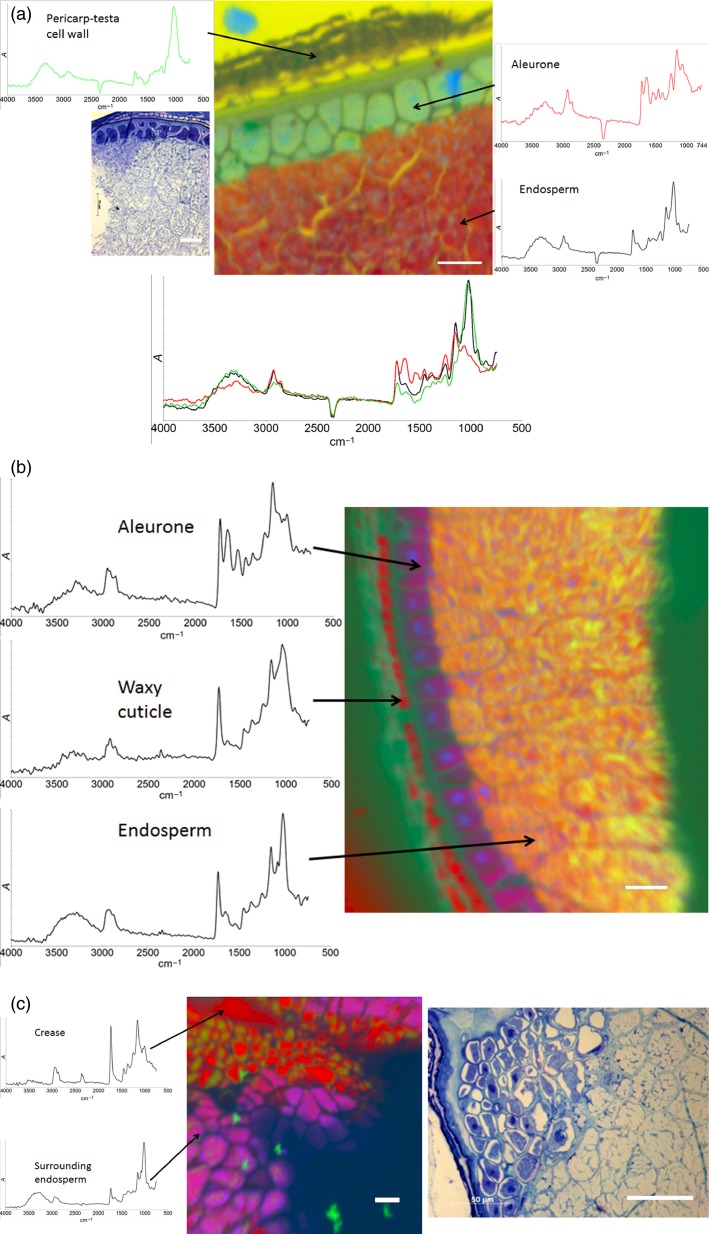
False-colour principal component analysis (PCA) images of sections through whole wheat kernels (Malacca and Consort), with representative spectra provided for regions of interest.(a) Section through the outer region of the wheat kernel, with a light-microscopy image of the same section, along with inset overlay spectra to allow for the comparison of peaks (see spectra at the bottom of the figure: black, endosperm; green, pericarp-testa; red, aleurone).(b) Section through the outer region of the wheat kernel (i.e. pericarp-testa, aleurone and endosperm).(c) Section through the crease region of the wheat kernel, with light-microscopy image of the same region. Scale bars: 50 μm.

The aleurone (Figure1b), which is the layer of cells below the pericarp-testa, shares a similar cell wall make–up, both structurally (shown by their spatial distribution in the image) and compositionally, as shown by the presence of similar peaks in the region 1000–1200 cm^−1^. The contents of the aleurone cells appear to be fairly uniform when imaged by ATR, and are predominantly composed of carbohydrate showing C–O stretching modes, although somewhat different in composition to the carbohydrate that makes up the cell wall. It seems likely that with the present samples the aleurone cell contents were not exposed, as they would be expected to have a more complex composition, including lipid, protein and inulin, as well as the presence of high concentrations of niacin and thiamine (Brouns *et al*., 2012). This is a limitation of the hand-dissection method of sample preparation, as the cell contents may not be exposed, or may be lost during dissection. Images for embedded and sectioned whole grains (Figure2) show more representative spectra for the aleurone cell contents.

The cells of the germ fraction show the most heterogeneous structure of any of the isolated wheat tissues (Figure1c). Germ tissue cells are rich in protein, as evidenced by the large amide–I and amide–II peaks. The amide–I peak (1600–1700 cm^−1^) arises from C=O stretching, and the amide–II peak (1510–1580 cm^−1^) derives from a combination of N–H bending and C–N stretching in the amide backbone of the protein molecules (Krimm and Bandekar, 1986), which are observed throughout the germ, associated with yellow regions in Figure1c. There are prominent lipid bodies, presumably consisting of oleosomes comprising mainly triacylglycerol, some of which may have coalesced to form larger lipid agglomerations (blue regions in Figure1c; Maurer *et al*., 2013). These show very strong absorbance peaks that are indicative of the presence of lipids, namely, the C–H stretch at 2800–3000 cm^−1^ and a peak from the ester linkage at 1750 cm^−1^.

Endosperm tissue also shows a highly heterogeneous structure. In Figure1(d), the cell walls of the endosperm cells are imaged in green, and individual starch granules can be seen as the small red spheres. Furthermore, protein bodies (pale blue) in the endosperm cells can be observed. The infrared spectrum of these pale-blue regions clearly shows the amide–I and amide–II peaks, indicative of a very high protein content in these regions.

The distinct chemical composition of each of the hand-dissected tissues of the wheat kernel was reflected in spectral differences observed through microspectral analysis of each dissected fraction. This clearly provides the possibility for the visualization, and potential quantification, of specific tissues within whole kernels using spectral differences between tissues.

### Whole wheat grain imaging

Sectioned and embedded wheat grains were also imaged to allow the differences between tissues to be visualized *in situ*. Figure2(a, b) shows the typical images obtained, along with light micrographs of one of the sections, clearly showing that different tissue types could be easily distinguished through PCA analysis of the spectra across an image. Similar spectra were obtained for the individual tissue types (endosperm, aleurone and pericarp-testa) present in Figure2(a, b) in whole embedded samples as were observed for the isolated tissues (Figures1a–d). Additionally, identifiable structures that are clearly characteristic of wheat tissue were observed in the sample sections. The imaging and sample preparation methods used in the present study, specifically using ATR with a Ge crystal and linear-array detector, along with the use of embedded samples that have been cut on a microtome to produce a flat surface on the submicron scale for imaging, allowed significantly improved resolution to be obtained, relative to previous studies (Miller and Dumas, 2006; Koç and Wetzel, 2007; Toole *et al*., 2007; Koç *et al*., 2008). This has allowed structural details such as cell walls, the waxy cuticle (Figure2b) and even individual starch granules to be visualized in the infrared images.

Figure2a shows light and infrared microspectroscopy images of a section through the outer layers of a wheat kernel. The endosperm tissue can be identified as the predominantly red-coloured region; this colour indicates a spectrum that matches that of starch granules closely. The red starch is interspersed with small blue regions, the spectra of which contain prominent amide absorption peaks (at ∼1650 and ∼1550 cm^−1^; Barth, 2007), suggesting that these are protein bodies. Surrounding the endosperm cells the outline of cell walls can clearly be seen in yellow, although it should be noted that in this particular image the endosperm cell walls have been infiltrated with resin, and give the same spectral signal as the background. The cells of the aleurone can be clearly seen in green. The spectra obtained from the aleurone cells reflect the complex make-up of these cells (Hemery *et al*., 2009), in that they contain a large number of peaks indicative of contributions from amide linkages (protein), non-starch polysaccharides and other carbohydrate components, in addition to distinct regions (blue) of protein bodies. The aleurone cell walls can clearly be visualized in dark green, and are resolved particularly distinctly in this image, with a resolution close to that achieved in the accompanying light-microscope image. The structure of the pericarp-testa can then be observed on the outside of the grain, with a spectrum that is dominated by non-starch polysaccharides, as would be expected for the pericarp-testa (Hemery *et al*., 2009). It should be noted that in this image no starch-related spectral signals were observed in the pericarp-testa, suggesting that the starch granules observed in Figure1(a) may be artifacts. A second high-resolution image of the outer layers of the wheat kernel (Figure2b) shows a similar level of structural resolution. In this case the sample embedding has preserved more of the structure of the sample. The endosperm cell walls are visible in purple, showing spectral similarity to the aleurone cell contents, presumably on account of protein bodies being adherent to the cell walls, resulting in a higher protein content. The waxy cuticle has also been preserved in the pericarp-testa layers in this image, and can clearly be seen in red, producing a spectrum with strong absorption bands at around 2900 cm^−1^ (methylene symmetric and anti-symmetric stretching modes; Safar *et al*., 1994) and 1730 cm^−1^ (C=O bond; Safar *et al*., 1994), indicative of the large quantity of lipid that is present in the waxy cuticle. Figure2(c) shows detail of the crease region, at the centre of the kernel, with a visible light micrograph of the same section shown for comparison. The lipid-rich cells that compose the bulk of the crease can be identified in red, and distinguished from the surrounding endosperm cells. Used together in this way, the combination of mid-infrared images combined with light micrographs can provide additional compositional information, something that light micrographs alone cannot easily achieve.

### Correlation analysis of whole grain images

**Figure 3:**
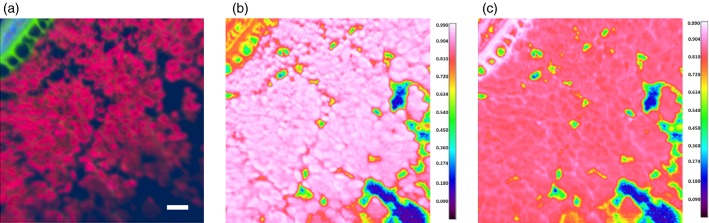
Outer region of the wheat kernel (Malacca and Consort), analysed by false-colour principal component analysis (PCA) and correlation analysis.(a) False-colour PCA image.(b) Correlation image using a spectrum of endosperm for comparison.(c) Correlation image using a spectrum of pericarp-testa for comparison. The ‘hot’ colours (white and red) in images (b) and (c) show regions with high correlation, whereas the ‘cool’ colours (blue and green) show areas of poorer correlation. Scale bars: 50 μm.

An alternative method that may be used to analyse hyperspectral images is correlation analyses. In the examples presented here, the spectra were extracted from the images obtained for purified pericarp-testa and endosperm (Figure1a, d, respectively) by averaging spectra across the complete image. This resulted in spectra that were representative of each tissue type. Regression analysis was then carried out using the representative spectra for each tissue type against hyperspectral images, and false-colour images were produced showing the correlation coefficient (*R*^2^ value) for each representative spectrum to each pixel in the hyperspectral image. In Figure3(a) the PCA false-colour image shows two distinct regions: green, which morphologically appears to be associated with aleurone cell walls and pericarp-testa; and pink, which morphologically appears to be endosperm tissue. Correlation analysis confirms this, with Figure3(b) showing the high correlation coefficient (as exhibited by ‘hot’ colours, whites and reds) of the endosperm regions, with an averaged spectrum taken from hand-dissected, purified endosperm, whereas the pericarp-testa and aleurone cell wall regions produced a very high correlation coefficient with the spectrum of purified pericarp-testa tissue (Figure3c). This analysis also highlights details in the endosperm, where cell wall material similar to the pericarp-testa can be more easily identified than in the PCA image (Figure3c).

**Figure 4:**
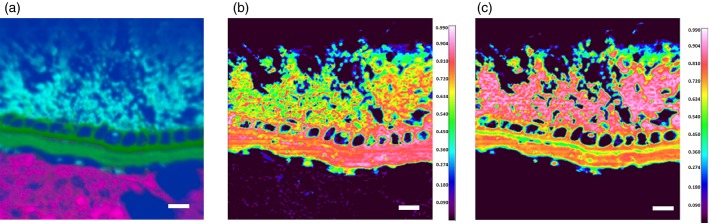
Outer region of the wheat kernel (Malacca and Consort), analysed by false-colour principal component analysis (PCA) and correlation analysis.(a) False-colour PCA image.(b) Correlation image using a spectrum of pericarp-testa for comparison.(c) Correlation image using a spectrum of endosperm for comparison. The ‘hot’ colours (white and red) in images (b) and (c) show regions with high correlation, whereas ‘cool’ colours (blue and green) show areas of poorer correlation. Scale bars: 50 μm.

Figure4 illustrates the utility of correlation analysis. The PCA false-colour image (Figure4a) allows the pericarp-testa to be identified morphologically, but there is material both below and above the pericarp-testa (in pink and sky blue, respectively) that yield spectra. The correlation analysis shown in Figure4(b), using a purified pericarp-testa reference spectrum, clearly highlights the cell walls of the pericarp-testa and aleurone in the centre of the image. Figure4(c), using a purified endosperm reference spectrum, identifies the ‘hot’, pink and white, regions as endosperm, whereas the bottom region of the image shows no correlation with any of the wheat spectra, indicating that the spectra observed in this region are artefacts arising from the spectrum of the embedding material. The same technique could be applied to identify a wide variety of sample contaminants from known reference spectra.

### Analysis of spectra obtained from images

**Figure 5:**
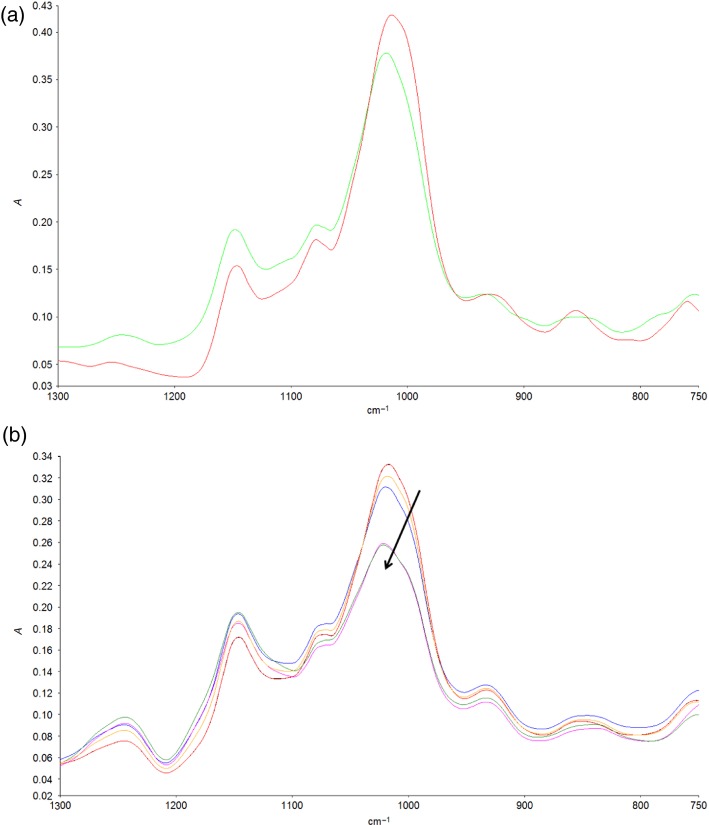
Averaged spectra from the starch-rich endosperm region of wheat images, normalized through standard normal variate (SNV).(a) Spectra from uncooked (red) and cooked (green) durum wheat samples.(b) Spectra from durum wheat samples during simulated gastric and duodenal digestion. The spectra are a digestion series, with an arrow indicating the direction of change in the spectra with increasing starch digestion time.

Images were captured of wheat endosperm samples collected before and after cooking (hydrothermal processing), and during a simulated *in vitro* digestion; from these images averaged spectra were obtained. Figure5(a) shows spectra of raw (red spectrum) and boiled (green spectrum) endosperm tissue following normalization (standard normal variate, SNV). The starch-associated peaks in the region 990–1020 cm^−1^ show a clear peak shift towards higher wave numbers as a result of the loss of structure that derives from starch gelatinization during hydrothermal processing. The changes observed in the starch peaks following heat treatment are the same as those observed in previous studies of the spectra of the *in situ* gelatinization of purified starch (Warren *et al*., 2013), and of the spectra of cooked and raw cereals (Cozzolino *et al*., 2014). This indicates that aspects of the physical structure of the food matrices can be determined on a micron scale through the application of infrared microscopy.

The cooked wheat samples were subjected to an *in vitro* digestion procedure, and were then imaged at different time points, from an undigested control (time zero), through to 30 min of gastric digestion, 30 min of gastric digestion plus 5 min of duodenal digestion, and 60 min of gastric digestion plus 20 min of duodenal digestion. Averaged and normalized (SNV) spectra for the starch peak during digestion are shown in Figure5(b). It can be clearly observed that the relative intensity of the starch peak at 990–1020 cm^−1^ decreases over time as the starch is digested by α–amylase during the *in vitro* digestion procedure, as has been observed previously in starch films (Snabe and Petersen, 2002). Thus, infrared microscopy is a tool that can be used to directly probe the intracellular digestion of starch in a complex food matrix, such as cooked wheat.

### Arabidopsis leaf imaging

**Figure 6:**
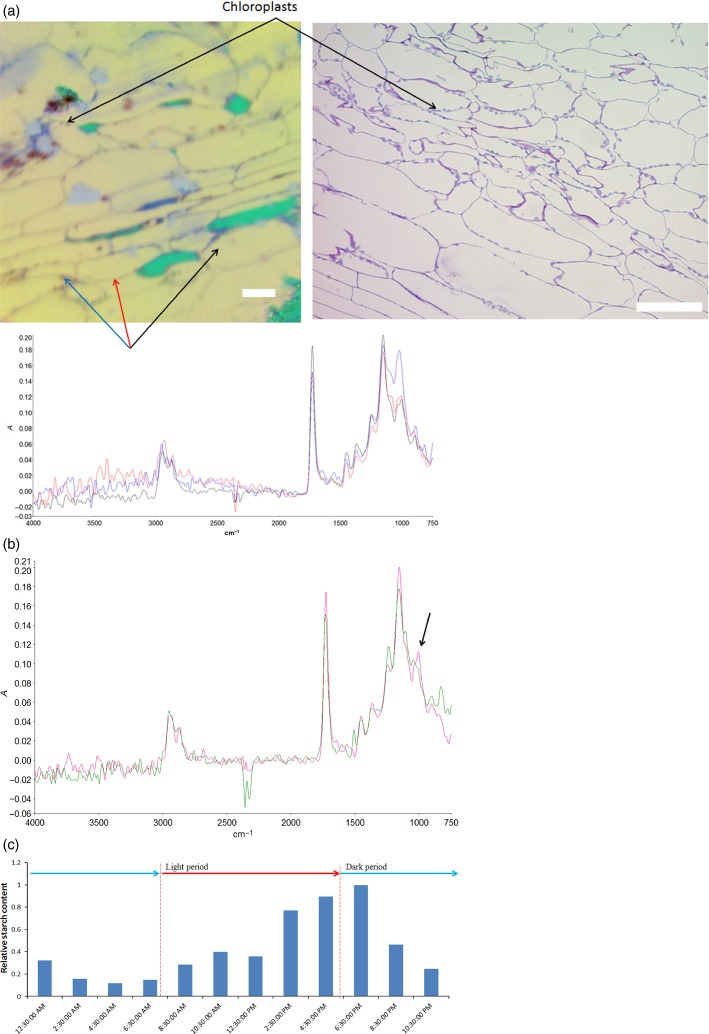
(a) False-colour principal component analysis (PCA) image of embedded Arabidopsis leaf. Cell walls and chloroplasts can be seen in black, whereas the cell contents are pale yellow. Green regions are cells that have emptied during preparation and have yielded no spectra. Spectra are shown from the cell contents (black spectra), cell walls (red spectra) and chloroplasts (blue spectra), as well as a light-microscopic image from a section cut from the same block, stained with toluidine blue (both scale bars 50 μm).(b) Spectra of the cell contents of embedded Arabidopsis leaves picked at 16:30 h (pink) and 08:30 h (green).(c) Relative starch concentrations in whole Arabidopsis plants measured through a complete diurnal period.

To further demonstrate the utility of microspectroscopy for the *in situ* identification and quantification of starch in plant tissues at a subcellular level, a study was carried out investigating diurnal variation of transient leaf starch in Arabidopsis. Figure S1(a, b) shows images for freshly sampled Arabidopsis leaves picked during the light period at 10:30 h (S1a) and at 12:30 h (S1b), imaged by placing the untreated leaf, as picked, directly under the ATR crystal. These images allow large structural features on the surface of the leaves, such as the leaf hairs (trichomes, in red) to be visualized, as well as finer details such as the outlines of some cell walls in light blue in Figure S1(b). The finer details of cell structures cannot be resolved, and the spectral signal from any possible differences in the chemical composition of the cell contents is masked by the presence of cell wall polysaccharides. Figure6(a) shows an image of an embedded and sectioned leaf (picked at 16:30 h, at the end of the light period). Fine details of the cell walls of the parenchyma cells can be observed, and close inspection and comparison with a light micrograph of the same leaf show that individual chloroplasts can be resolved adhering to the cell walls. Inspection of the spectra of the chloroplasts in comparison with the spectra of the cell contents, and the surrounding cell walls, show a prominent peak at around 1000 cm^−1^ in the chloroplasts, which is much less intense in the spectra of surrounding tissue, arising from the high concentration of starch found in the chloroplast. Comparing spectra from the interior of parenchyma cells from embedded and sectioned leaves picked at 16:30 and 08.30 h (Figure6b, start of the light period) shows a distinct peak at around 1000 cm^−1^ arising from the accumulation of carbohydrate in the cell in the leaf picked at 16:30 h, which is completely absent in the spectra of cells from leaves picked at 08:30 h. As can be seen from the overall starch contents shown in Figure6(c) at different time points, the presence of a starch peak in cells at 16:30 h accords with peak starch deposition, whereas the absence of the peak at 08:30 h correlates with the lowest measured starch content, at the end of the dark period.

## Discussion

Infrared microspectroscopy is a method that has been used extensively to study the chemistry and functional properties of wheat, as well as other crop plants. Notably, it has been used in applications as varied as the study of protein structure (Wetzel *et al*., 2003), measurements of microstructural chemical features to inform programmes of research involved with plant breeding (Yu *et al*., 2004), exploring the molecular basis of low-temperature tolerance (Yu *et al*., 2005) and for exploring rhizome structure in legumes (Raab and Martin, 2001). Infrared microspectroscopy has a number of advantages over conventional analytical methods for analysing plant tissues. Chemical methods of analysis are destructive and fail to capture the fine details of the cellular structure of plant materials. Introducing the ability to identify the chemical composition of plant tissue with cellular level spatial resolution significantly enhances sensitivity. Thus, very minor components that are spatially separated in the intact tissue to individual cells or cell layers (e.g. aleurone) can be identified, and significantly less material is required for imaging analysis than is required for conventional chemical analysis. In addition, the spatial resolution afforded by infrared microscopy allows changes, as may occur, for example, in plant breeding studies, to be identified in the distribution of components within individual tissues, which could not be resolved with conventional chemical analysis. Conventional microscopy approaches allow details of cellular structure to be observed with a high resolution, but rely on stains to provide contrast, limiting the quantity of information that can be obtained about the chemical composition of different structures, as well as being highly time-consuming and requiring expert microscopists. Contrast between different chemical components in infrared images can be obtained through spectral analysis, meaning that there is no need to stain samples, and information about chemical composition may be obtained directly.

In the present study, two separate approaches were employed to analyse the spectral data in the images: PCA and correlation analysis. This is in contrast to previous studies, which have predominantly focused on the analysis of individual absorption peaks or peak ratios (Yu *et al*., 2004, 2005; Barron *et al*., 2005). The two approaches described here can yield contrasting information. PCA is an unsupervised form of data analysis, which produces images on the basis of statistical differences between the spectra representative of individual pixels. This allows contrast to be obtained between many different chemical components in an individual image: for example, in Figure2(a) structures can be identified as starch-containing material, protein bodies, aleurone cells, cell walls and the background embedding resin. Identifying such a wide variety of structures in a complex sample such as wheat tissue would be extremely difficult from an analysis of individual absorption bands, especially because many bands can be present in different structures. Additionally, false-colour PCA analysis can reveal structures that the experimenter is not, *a priori*, expecting to see in the sample. For example, without PCA, our observation of starch granules in pericarp tissue (Figure1c) could easily have been mistaken for unusual cell wall structures, and therefore incorrectly identified as an artefact.

False-colour PCA alone cannot, however, directly provide information about the composition of different structures within samples without a combination of analyses of individual spectra, and observations of the morphology of the sample, although it is useful for generating contrast between structures differing in composition. An alternative method, correlation analysis, as demonstrated in Figures3 and 4, uses spectra obtained from the purified components of the wheat kernel to identify specific structures. This is a very powerful method as it allows the chemical composition of any structure within an image to be identified, merely by obtaining a purified sample, without any need for further steps (e.g. stains or fluorescent antibodies). Spectra of purified components may be obtained from pure chemicals, mixtures of known composition or isolated components, as in the present example, and allows for label-free identification of these components within images.

The Arabidopsis images highlight the improvements in resolution that may be obtained through careful sample preparation prior to microspectroscopic imaging. Placing samples directly under the ATR crystal allows for the identification of gross structural features at the scale of 100s of μm, but produces images that are of limited use for identifying cell-by-cell variation. Fixing, embedding and sectioning the leaves prior to microspectroscopic imaging allows a far greater degree of detail to be identified, down to the scale of individual chloroplasts. The spectral signal resulting from starch accumulation in the plant can be clearly identified, despite the presence of penetrating resin (the spectral signal of which is removed at the pre-processing step of the data analysis). The ability to identify and quantify starch deposition on a cell-by-cell basis has clear applications for the study of starch synthetic enzyme mutants, where differential starch deposition between different cell types has been inferred from light and electron microscopic investigation (Pfister *et al*., 2014), but chemical methods to identify starch on a cell-by-cell basis are not currently available.

The strength of infrared imaging methods when compared with conventional bulk infrared approaches is that chemical information may be obtained with spatial resolution. Figure5 gives two examples of the kind of information that can be obtained, showing the distinct difference between cooked and raw starch, and the loss of starch during *in vitro* digestion using a model gut system (Wickham *et al*., 2012). Thus, future work could focus in more detail on using these spectral differences to observe spatial differences in the degree of starch gelatinization, or the degree of starch digestion within a complex plant food matrix, as has been observed for lipid digestion using more conventional methods (Ellis *et al*., 2004; Mandalari *et al*., 2008).

We are confident that the method we describe has many advantages over the methods in current use, but it is important that any limitations of the technique are recognized. As discussed in the introduction, the wavelength of infrared radiation places a fundamental limit on the resolution that can be obtained through infrared imaging of ∼5–7 μm. The computational data analysis methods used in the present paper allow structures below this limit to be resolved, down to ∼1.5–2.0 μm in size, but it seems unlikely that significant improvements beyond this are feasible. This limits the method to larger subcellular structures (starch granules, chloroplasts, etc.), and precludes studying the chemical composition of individual cells at the subcellular level. Although an advantage for IR microscopy is that there is no need for sample preparation, in the present work it was shown that there is a need to carry out sample preparation to achieve the highest possible resolution. Indeed, detailed, careful and time-consuming sample preparation is well justified by the need to preserve the fine details of anatomical structures. Presenting such intact structures to the ATR crystal allows them to be imaged.

## Conclusion

The present study illustrates the application of high-resolution infrared microspectroscopy to investigate the microstructure of wheat kernels and Arabidopsis leaves. The images presented in the current work represent a higher resolution than has previously been obtainable with bench-top instrumentation for the visualization of wheat kernels, allowing detailed anatomical structures to be observed directly in the infrared images. Two analysis methods are employed to allow structures to be differentiated. False-colour PCA images allow a wide range of structures to be identified within a single image, with no *a priori* knowledge of a sample's composition. Correlation analysis allows individual components to be identified with images in a label-free manner, using spectra from isolated or purified components. Further information regarding the microstructure and chemical composition of the samples can be obtained through the detailed analysis of the large volume of spectral information generated for each image. This approach, using high-spatial resolution, has considerable potential for determining the physicochemical changes in foods during mechanical and heat processing, and also when foods are broken down during mammalian digestion. The application of this technique could be even more wide-ranging, e.g. in forensic analysis, plant breeding studies and food quality control.

## Experimental Procedures

Hard winter wheat varieties (Malacca and Consort varieties of *Triticum aestivum* L.) were carefully dissected by hand using a scalpel and a microscope at the University of Manchester Satake Centre for Grain Process Engineering (UK), using the technique described in detail by Choomjaihan (2008). The dissection technique produced four fractions of the wheat caryopsis, namely the endosperm, aleurone, pericarp-testa layers and the germ (the embryo-rich fraction).

Durum wheat grains (*Triticum durum* Desf., Svevo cv.) were subjected to abrasion (2 min) in a Satake TM05 debranner (Satake, http://www.satake-group.com) to remove the majority of the outer layers (i.e. pericarp-testa and aleurone), and were subsequently milled (Satake STR-100 test-roller mill equipped with 4.13 fl cm^−1^ break-rolls) and sieved to select coarse macroparticles of 1.7–2.1 mm in diameter. The durum wheat macroparticles were prepared by hydrothermal processing in deionized water at 100°C (20 min), and then subjected to simulated oral exposure (using a laboratory preparation of salivary fluid and salivary α–amylase) followed by gastric digestion for up to 60 min and duodenal digestion for up to 20 min using the Dynamic Gastric Model (DGM) at The Institute for Food Research, Norwich, UK, as described in detail previously (Pitino *et al*., 2010; Vardakou *et al*., 2011; Wickham *et al*., 2012). Samples were collected at various stages of digestion and immediately immersed in Karnovsky's fixative (1.6% formaldehyde and 2% glutaraldehyde in 0.08 m sodium cacodylate, pH 7.2) to prevent further degradation. Fixed samples were then rinsed in 0.1 m sodium cacodylate buffer, dehydrated in increasing concentrations of ethanol and infiltrated with Spurr low-viscosity resin (EM0300; Sigma-Aldrich, http://www.sigmaaldrich.com) using propylene oxide as a transition solvent. After 1 week, resin-embedded samples were cured at 70 ± 2°C for 12 h and then sectioned (0.5–μm thickness) on an Ultracut E microtome fitted with a glass knife (Reichert-Jung, now Leica, http://www.leica.com). Sections were stained with 0.1% toluidine blue, and viewed with a Leica Axioskop MOT plus microscope with an Axiocam MRc digital camera for image acquisition. The sample blocks remaining after sectioning were used for ATR imaging.

*Arabidopsis thaliana* plants (ecotype Columbia) were grown in soil under a short-day regime (8^ ^h light/16–h dark). Two leaves were selected from the fully expanded rosette leaves at stage 3.9, as described by Boyes *et al*. (2001). These fresh leaves were then used for ATR imaging. Embedded leaves were picked at the same growth stage and immersed immediately in glutaraldehyde (3%, v/v) in Sorenson's phosphate buffer (pH 7.2; cat. no. 16539–45; Electron Microscopy Sciences, http://www.emsdiasum.com) to prevent degradation. Fixed samples were then rinsed in Sorenson's phosphate buffer (0.133 m Na_2_HPO_4_, 0.133 m KH_2_PO_4_; pH 7.2). Subsequent dehydration and resin-embedding were performed as described previously. The sample blocks were used for ATR imaging.

To determine the starch content of the leaves at several time points over the diurnal period, three plants at stage 3.9 were collected and pooled every 2 h. These plants were flash frozen in liquid nitrogen and cryogenically ground. The starch was extracted and dissolved in a DMSO solution containing lithium bromide (0.5% w/w; DMSO/LiBr), as described in Powell *et al*. (2014). This solution was subsequently analysed using the Megazyme Total Starch assay kit (Megazyme, http://www.megazyme.com) to determine the level of starch at each time point. For the analysis of total starch, a 0.2–ml aliquot of sample from the initial extraction, dissolved in 0.5 ml DMSO/LiBr, was transferred into a pre-weighed centrifuge tube and the precise sample weight (SW) was recorded. The sample was incubated with 0.3 ml of thermostable α–amylase solution [50 mm 3–(*N*–morpholino)propanesulphonic acid (MOPS), pH 7.0; Megazyme] in a boiling water bath for 12 min with stirring. Sodium acetate buffer (0.49 ml, 200 mm, pH 4.5) and 10 μl of amyloglucosidase (Megazyme) were added to the solution, which was then incubated at 50°C with shaking for a further 30 min. The final weight (FW) of the whole solution was recorded and the samples were subsequently centrifuged at 4000 ***g*** for 10 min. A 0.1–ml aliquot of the supernatant was transferred to the centrifuge tube and 3.0 ml of GOPOD reagent [*p*–hydroxybenzoic acid and sodium azide (0.4% w/v), glucose oxidase plus peroxidase and 4–aminoantipyrine; Megazyme, Wicklow, Ireland] was transferred to the tube. The samples were incubated at 50°C with occasional shaking for 20 min. A glucose control (0.1 ml d–glucose standard, 1 mg ml^−1^) and a blank control (0.1 ml of distilled water) were also prepared and incubated with 3.0 ml of GOPOD reagent for 20 min. The absorbance of the samples and glucose standard were read against the blank control at 510 nm, and the concentration of starch in DMSO/LiBr (mg ml^−1^) was calculated as follows:

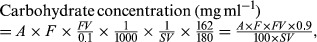
1

where *A* is the absorbance read against the blank control, *F* is the conversion from absorbance to μg, *FV* is the final volume of the sample, 0.1 is the volume size of the diluted sample being analysed, 1/1000 is the conversion from μg to mg, *SV* is the initial sample volume and 162/180 is the conversion from free d–glucose to anhydro-d-glucose, as in carbohydrate.

All spectroscopic images were obtained using a Spotlight 400 microscope fitted with a Ge ATR crystal (PerkinElmer, http://www.perkinelmer.com), attached to a Frontier spectrometer (PerkinElmer). The detector used in this system is a liquid nitrogen-cooled, 16–element mercury–cadmium–telluride linear array. The Fourier transform instrument is operated in rapid scan mode and the image is obtained by translating the ATR crystal and sample. A spectral resolution of 16 cm^−1^ was used with an interferometer scanning speed of 1 cm sec^−1^. An imaging area of either 300 × 300 μm for smaller images or 500 × 500 μm for larger images was selected, with an image pixel size of 1.56 μm (corresponding to an oversampling factor of two, compared with the diffraction-limited spatial resolution). For a 500 × 500 μm image this results in an image composed of more than 102 000 individual spectra, with a total data acquisition time of between 3 and 5 h, depending on image size. A range of approaches may be used to analyse the hyperspectral data cubes obtained, including supervised analysis methods to identify specific chemical compounds, or combinations of compounds, analogous to using a specific stain, or combination of stains, in light microscopy, and unsupervised analysis methods that provide an overview of the chemical variation across a sample. Following image acquisition, a false-colour image of the chemical composition of each sample was generated through PCA of the obtained spectra using the ‘Show structure’ function built into SpectrumImage. This function performs some pre-processing of the image cube (to remove uninformative baseline variations), followed by a principal components decomposition. The display colour for each pixel is then obtained by mapping the first three principal component scores onto the red, green and blue colour values of the pixel. Because the first few principal components are likely to contain contributions from many or all of the structural factors in the sample, this approach often results in a unique colour being assigned to each tissue type, and is far more informative than simply displaying average absorbance or individual principal component scores as false-color images. Although ‘Show structure’ is effective at revealing spatial structure in the image, it does not provide information about the actual identity of the structures revealed. This task was achieved by using the ‘Compare correlation’ function of SpectrumImage to produce images presenting the correlation between the image pixels and spectra obtained independently from isolated wheat tissues. This function analyses the similarity between a defined reference spectrum and the spectra in a hyperspectral data cube, calculating an *R*^2^ value for each individual spectrum in the hyperspectral data cube, and plotting those *R*^2^ values as a heat map, indicating regions of the sample that have a high similarity to the reference spectrum.

## Supporting Information

**Figure S1.** Arabidopsis leaf architecture, imaged by placing the leaf directly underneath the ATR crystal with no sample preparation.
