# Supplementary material for: Infrared microspectroscopic imaging of plant tissues: spectral visualization of Triticum aestivum kernel and Arabidopsis leaf microstructure
Source: Plant J. 2015 Oct 23;84(3):634–46. doi: 10.1111/tpj.13031 (PMC4620737; doi:10.1111/tpj.13031)
Supplement: Supplementary file 2 [file tpj0084-0634-sd2.docx]

**Supporting Information Legend**

**Figure S1.** Arabidopsis leaf architecture, imaged by placing the leaf directly underneath the ATR crystal with no samples preparation. Red structures are trichomes. a. leaf picked at 10.30am; b. leaf picked at 12.30pm
